# Extending the PRISMA statement to equity-focused systematic reviews (PRISMA-E 2012): explanation and elaboration

**DOI:** 10.1186/s12939-015-0219-2

**Published:** 2015-10-08

**Authors:** Vivian Welch, Mark Petticrew, Jennifer Petkovic, David Moher, Elizabeth Waters, Howard White, Peter Tugwell, the PRISMA-Equity Bellagio group

**Affiliations:** Bruyere Research Institute, 43 Bruyère St, Annex E, room 304, Ottawa, K1N 5C8 Ontario Canada; LSHTM, 15-17 Tavistock Place, London, WC1H 9SH UK; Research Associate, 43 Bruyère St, Annex E, room 304, Ottawa, K1N 5C8 Ontario Canada; Ottawa Hospital Research Institute, The Ottawa Hospital, General Campus, Centre for Practice Changing Research Building 501 Smyth Road, PO BOX 201B, Ottawa, Ontario K1H 8L6 Canada; School of Population and Global Health, University of Melbourne, Level 5, 207 Bouverie Street, Victoria, 3010 Australia; International Initiative for Impact Evaluation (3ie), Global Development Network, Post Box No. 7510, Vasant Kunj PO, New Delhi, India; Department of Medicine, University of Ottawa, 43 Bruyère St, Annex E, room 304, Ottawa, K1N 5C8 Ontario Canada

## Abstract

**Background:**

The promotion of health equity, the absence of avoidable and unfair differences in health outcomes, is a global imperative. Systematic reviews are an important source of evidence for health decision-makers, but have been found to lack assessments of the intervention effects on health equity. The Preferred Reporting Items for Systematic Reviews and Meta-Analysis (PRISMA) is a 27 item checklist intended to improve transparency and reporting of systematic reviews. We developed an equity extension for PRISMA (PRISMA-E 2012) to help systematic reviewers identify, extract, and synthesise evidence on equity in systematic reviews.

**Methods and findings:**

In this explanation and elaboration paper we provide the rationale for each extension item. These items are additions or modifications to the existing PRISMA Statement items, in order to incorporate a focus on equity. An example of good reporting is provided for each item as well as the original PRISMA item.

**Conclusions:**

This explanation and elaboration document is intended to accompany the PRISMA-E 2012 Statement and the PRISMA Statement to improve understanding of the reporting guideline for users. The PRISMA-E 2012 reporting guideline is intended to improve transparency and completeness of reporting of equity-focused systematic reviews. Improved reporting can lead to better judgement of applicability by policy makers which may result in more appropriate policies and programs and may contribute to reductions in health inequities.

To encourage wide dissemination of this article it is accessible on the International Journal for Equity in Health, Journal of Clinical Epidemiology, and Journal of Development Effectiveness web sites.

**Electronic supplementary material:**

The online version of this article (doi:10.1186/s12939-015-0219-2) contains supplementary material, which is available to authorized users.

## Introduction

Promoting health equity and reducing avoidable health inequalities is a global imperative, endorsed by the Rio Summit in Brazil in 2011, the Pan American Health Organization, and the World Health Organization [1–3]. Health inequalities are differences in health outcomes across individuals in a population or between different population groups whereas health inequities are inequalities which are avoidable and unfair [4, 5]. Inequities are not only due to poverty, but may also be due to unfair differences in health across other characteristics such as sex/gender, geography, and ethnicity [6]. The concept of health equity also suggests that groups of people should not be prevented from achieving health due to factors such as discrimination or inadequate access. In this reporting guideline, we focus on unfair inequalities in health outcomes and therefore use the term ‘equity’.

Systematic reviews are recognized as an important source of rigorously and transparently synthesized information by health decision-makers [2, 7–9]. Health decision makers have described lack of evidence on equity as a barrier to using systematic reviews and guidelines [5, 10], and arguably, primary studies themselves. However, a 2010 systematic review found that there is a lack of detail in reporting of certain aspects important to health equity including population characteristics, assessment of credibility of subgroup analyses, and judgement about the applicability of the findings to other settings with fewer than half of the included reviews reporting on socio-demographic characteristics (such as age, sex, place of residence, ethnicity) of the study populations [11]. These are important factors to consider for health equity and the lack of reporting of these elements demonstrates the need to improve reporting of equity in systematic reviews, and to increase the overall investment in systematic reviews that can provide a clear emphasis on considerations of equity.

Reporting guidelines have been shown to improve reporting of different study designs [12, 13]. The Preferred Reporting Items for Systematic Reviews and Meta-Analyses (PRISMA) is a 27 item checklist to ensure complete and transparent reporting of the methods used in systematic reviews [14]. However, the original PRISMA Statement did not include items specific for reporting on considerations of equity. Equity considerations include the definition of disadvantaged populations, methods to include equity considerations in analyses, and applicability of the evidence to other settings or populations. We developed an equity extension of the PRISMA Statement, called PRISMA-E 2012 to respond to these needs [15]. As of July 8, 2015, the PRISMA-E 2012 reporting guideline has been viewed almost 16,000 times, downloaded 2661 times, cited 50 times (Scopus), and shared 109 times using Twitter (99 tweets by 70 users according to Altmetrics). It is also cited as a reference for the World Health Organization Handbook on Guideline Development, the Oxford Textbook of Public Health, the Public Health Agency of Canada guidance, the Canadian Institutes of Health Research instructions for applicants and the Journal of the Society for Social Work and Research. The Spanish version of PRISMA-E 2012, published in July 2013, has been downloaded 477 times as of November 17, 2014 (Scielo) and has received 1474 visits on the Journal’s website [16].

**Box 1:** **Terminology related to disadvantaged populations**

To describe the populations who are experiencing inequitable differences we use the term ‘disadvantaged’ although we recognize that this term may not be acceptable to all. In a methodology review of equity assessment, disadvantage was defined in terms of the avoidability or preventability of health inequalities (12 of 34 studies) [11] by focusing on populations that have experienced health inequities (e.g., Aboriginal populations).
We have chosen to use the term “disadvantaged” for PRISMA-E 2012 because we felt that despite its limitations (e.g., that it may be considered a condescending or paternalistic term), the term “disadvantaged” more clearly defines a population that is experiencing or has experienced health inequities. Whereas vulnerability encompasses a combination of risk, exposure and resilience that do not always lead to health inequities, and other terms such as “marginalized” are too narrowly focused and do not encompass the breadth of settings, contexts and health inequities of interest.

To further facilitate and promote the use of the guideline of equity issues in systematic review (PRISMA-E 2012), we developed this explanation and elaboration to describe each of the items and provide examples from existing reviews to demonstrate good reporting.

### Scope of PRISMA-E 2012

The PRISMA-E 2012 checklist was developed to improve transparency and completeness of reporting of systematic reviews of intervention studies with a focus on health equity. We define systematic reviews of intervention studies with a major focus on health equity as those designed to:

1. Assess effects of interventions targeted at disadvantaged or at-risk populations (e.g., school feeding for disadvantaged children [17]). These may not include equity outcomes but by targeting disadvantaged populations will reduce inequities.
2. Assess effects of interventions aimed at reducing social gradients across populations or among subgroups of the population (e.g., interventions to reduce the social gradient in smoking, obesity prevention in children, interventions delivered by lay health workers [15, 18–20]).

In the PRISMA-E 2012 Statement we had a third type of systematic review focused on health equity, those that are not aimed at reducing inequities but where it may be important to understand the equity effects. For example, we had previously categorized the review examining lay health workers in this category. We have now grouped this review into the second type of review described above.

In 2010, approximately 20 % of systematic reviews indexed in Medline met at least one of the above criteria [21, 22]. These reviews may not include equity as an outcome, but may target disadvantaged populations, or assess differences of the effect of the intervention among disadvantaged populations.

The PRISMA-E 2012 items are focused on health equity but may also apply to systematic reviews in non-health areas which address questions about inequity such as education, transport, justice, or social welfare. Additionally, some items in the checklist may be relevant to all systematic reviews, but have been included in this extension because of their specific importance to health equity. These items are additions or modifications to the existing PRISMA Statement items, in order to incorporate a focus on equity. For each item, the original PRISMA item is listed and the PRISMA-E 2012 extension item is noted below.

## Methods PRISMA-E 2012 reporting guideline

To develop the PRISMA-E 2012 reporting guideline, we followed the series of steps recommended by Moher and colleagues (2010), as reported in the previously published paper [23]. The first step was to identify need and review the literature. We conducted a systematic review and a methodological study [22, 24]. Next, we conducted an online survey whose respondents included systematic review authors, policy makers, and systematic review funders [15]. Finally, we held a consensus meeting of international experts from February 9–10, 2012 at the Rockefeller Foundation’s Bellagio Conference Centre in Bellagio, Italy. We took detailed minutes at the meeting and used these minutes to revise the PRISMA-E statement and develop this explanation and elaboration document. The complete PRISMA-E 2012 checklist is provided in Table 1.

**Table 1:** Checklist of items for reporting equity-focused systematic reviews

Section	Item	Standard PRISMA Item	Extension for Equity-Focused Reviews
Title			
Title	1	Identify the report as a systematic review, meta-analysis, or both.	Identify equity as a focus of the review, if relevant, using the term equity.
Abstract			
Structured summary	2	2. Provide a structured summary including, as applicable: background; objectives; data sources; study eligibility criteria, participants, and interventions; study appraisal and synthesis methods; results; limitations; conclusions and implications of key findings; systematic review registration number.	State research question(s) related to health equity.
	2A		Present results of health equity analyses (e.g., subgroup analyses or meta-regression).
	2B		Describe extent and limits of applicability to disadvantaged populations of interest.
Introduction			
Rationale	3	Describe the rationale for the review in the context of what is already known.	Describe assumptions about mechanism(s) by which the intervention is assumed to have an impact on health equity.
	3A		Provide the logic model/analytical framework, if done, to show the pathways through which the intervention is assumed to affect health equity and how it was developed.
Objectives	4	Provide an explicit statement of questions being addressed with reference to PICOS.	Describe how disadvantage was defined if used as criterion in the review (e.g., for selecting studies, conducting analyses, or judging applicability).
	4A		State the research questions being addressed with reference to health equity
Methods			
Protocol and registration	5	Indicate if a review protocol exists, if and where it can be accessed (e.g., web address), and, if available, provide registration information including registration number.	
Eligibility criteria	6	6. Specify study characteristics (e.g., PICOS, length of follow-up) and report characteristics (e.g., years considered, language, publication status) used as criteria for eligibility, giving rationale.	Describe the rationale for including particular study designs related to equity research questions.
	6A		Describe the rationale for including the outcomes (e.g., how these are relevant to reducing inequity).
Information sources	7	Describe all information sources (e.g., databases with dates of coverage, contact with study authors to identify additional studies) in the search and date last searched.	Describe information sources (e.g., health, non-health, and grey literature sources) that were searched that are of specific relevance to address the equity questions of the review.
Search	8	Present full electronic search strategy for at least one database, including any limits used, such that it could be repeated.	Describe the broad search strategy and terms used to address equity questions of the review.
Study selection	9	State the process for selecting studies (i.e., screening, eligibility, included in systematic review, and, if applicable, included in the meta-analysis).	
Data collection process	10	Describe method of data extraction from reports (e.g., piloted forms, independently, in duplicate) and any processes for obtaining and confirming data from investigators.	
Data items	11	List and define all variables for which data were sought (e.g., PICOS, funding sources) and any assumptions and simplifications made.	List and define data items related to equity, where such data were sought (e.g., using PROGRESS-Plus or other criteria, context).
Risk of bias in individual studies	12	Describe methods used for assessing risk of bias of individual studies (including specification of whether this was done at the study or outcome level), and how this information is to be used in any data synthesis.	
Summary measures	13	State the principal summary measures (e.g., risk ratio, difference in means).	
Synthesis of results	14	Describe the methods of handling data and combining results of studies, if done, including measures of consistency (e.g., *I* ^2^) for each meta-analysis.	Describe methods of synthesizing findings on health inequities (e.g., presenting both relative and absolute differences between groups).
Risk of bias across studies	15	15. Specify any assessment of risk of bias that may affect the cumulative evidence (e.g., publication bias, selective reporting within studies).	
Additional analyses	16	Describe methods of additional analyses (e.g., sensitivity or subgroup analyses, meta-regression), if done, indicating which were pre-specified.	Describe methods of additional synthesis approaches related to equity questions, if done, indicating which were pre-specified
Results			
Study selection	17	Give numbers of studies screened, assessed for eligibility, and included in the review, with reasons for exclusions at each stage, ideally with a flow diagram.	
Study characteristics	18	For each study, present characteristics for which data were extracted (e.g., study size, PICOS, follow-up period) and provide the citations.	Present the population characteristics that relate to the equity questions across the relevant PROGRESS-Plus or other factors of interest.
Risk of bias within studies	19	Present data on risk of bias of each study and, if available, any outcome level assessment (see item 12).	
Results of individual studies	20	For all outcomes considered (benefits or harms), present, for each study: (a) simple summary data for each intervention group; (b) effect estimates and confidence intervals, ideally with a forest plot.	
Synthesis of results	21	Present results of each meta-analysis done, including confidence intervals and measures of consistency.	Present the results of synthesizing findings on inequities (see 14).
Risk of bias across studies	22	Present results of any assessment of risk of bias across studies (see item 15).	
Additional analysis	23	Give results of additional analyses, if done (e.g., sensitivity or subgroup analyses, meta-regression [see item 16]).	Give the results of additional synthesis approaches related to equity objectives, if done, (see 16).
Discussion			
Summary of evidence	24	Summarize the main findings including the strength of evidence for each main outcome; consider their relevance to key groups (e.g., health care providers, users, and policy makers).	
Limitations	25	Discuss limitations at study and outcome level (e.g., risk of bias), and at review-level (e.g., incomplete retrieval of identified research, reporting bias).	
Conclusions	26	Provide a general interpretation of the results in the context of other evidence, and implications for future research.	Present extent and limits of applicability to disadvantaged populations of interest and describe the evidence and logic underlying those judgments.
	26A		Provide implications for research, practice, or policy related to equity where relevant (e.g., types of research needed to address unanswered questions).
Funding			
Funding	27	Describe sources of funding for the systematic review and other support (e.g., supply of data); role of funders for the systematic review.	

This checklist should be read in conjunction with the Statement and Explanation and Elaboration document

*PICOS* participants, interventions, comparisons, outcomes, and study design

### How to use this paper

The format of this document is similar to the format used in other explanation and elaboration documents [25–29]. We feel this explanation and elaboration paper is an important contribution to the literature because it provides the detailed rationale, evidence, whenever available, and an exemplar, for recommending each item as well as examples of good practice. We recommend authors use this document in conjunction with the PRISMA-E 2012 statement and with the original PRISMA statement and explanation and elaboration papers. We use the term “we” to refer to the consensus panel that met to finalize the PRISMA-Equity 2012 reporting guidelines in February 2012, as well as those who were unable to attend but contributed to the final reporting guidelines.

#### Item 1: title

*Standard PRISMA item: Identify the report as a systematic review, meta-analysis or both.*

*In addition, for equity-focused systematic reviews: Identify equity as a focus of the review, if relevant, using the term equity*

Examples

- **○** “Inequity in childhood immunization in India: a systematic review” [30]
- **○** “Can cost-effectiveness analysis integrate concerns for equity? Systematic review” [31]

Explanation - Equity-focused systematic reviews need a concise title that includes the term ‘equity’ or ‘inequity’. At the consensus meeting, the panel felt strongly that a consistent term was needed in the title to help identify equity-focused reviews and we chose the term ‘equity’ because of our focus on unfair inequalities in health. Indexing of electronic databases is poor for terms relating to health equity or disadvantaged or vulnerable populations therefore we suggest including ‘equity’ in the title will facilitate searching for equity-focused reviews. Not all systematic reviews will include equity in the title so to improve searchability, ‘equity’ should be included in the abstract and/or keywords. This will also help policy makers find equity-focused systematic reviews. In a search of systematic reviews published in the last year in MEDLINE, we only found 11 with ‘equity’ in the title and 73 with equity in the title and/or abstract (Additional file 1: Table S1).

#### Item 2: abstract

*Standard PRISMA item: Provide a structured summary including, as applicable: background; objectives; data sources; study eligibility criteria, participants, and interventions; study appraisal and synthesis methods; results; limitations; conclusions and implications of key findings; systematic review registration number.*

*In addition, for equity-focused systematic reviews: State research question(s) related to health equity.*

Example

- **○** “We aimed to systematically assess current evidence for the association between socioeconomic position (SEP) and caries. We included studies investigating the association between social position (determined by own or parental educational or occupational background, or income) and caries prevalence, experience, or incidence” [32].
- “Our primary outcome is the utilization of [post-natal care] PNC services, and determinants of concern are: 1) socioeconomic status (for example, income, education); 2) geographic determinants (for example, distance to a health center, rural versus urban residence); and 3) demographic determinants (for example, ethnicity, immigration status)” [33]

Explanation - The abstract of the review needs to indicate whether the research questions and objectives are of relevance to equity or specific populations since some readers, including those making decisions about health programs and policies, may only have access to the abstract (or only read the abstract). Thus, we recommend research questions related to health equity should be reported in the abstract to facilitate their retrieval for decision-making. We also recommend describing the type of inequities addressed by the review (e.g., health outcomes, health service coverage or access, financial risk).

*Item 2A: in addition, for equity-focused systematic reviews: Present results of health equity analyses (e.g., subgroup analyses or meta-regression).*

Example

- **○** “No strong evidence of differential effects was found for smoking restrictions in workplaces and public places, although those in higher occupational groups may be more likely to change their attitudes or behaviour. Smoking restrictions in schools may be more effective in girls. Restrictions on sales to minors may be more effective in girls and younger children. Increasing the price of tobacco products may be more effective in reducing smoking among lower income adults and those in manual occupations, although there was also some evidence to suggest that adults with higher levels of education maybe more price sensitive. Young people aged under 25 are also affected by price increases, with some evidence that boys and non-white young people may be more sensitive to price” [18].

Explanation - Findings related to equity questions should be presented in the abstract along with the main results. In addition, the abstract needs to differentiate between the main analyses and other analyses as well as any null findings. Of 182 abstracts, 42 % do not describe the direction of the main effect in words, and 25 % do not provide numerical results [34]. For equity-focused reviews, we have found that equity findings (e.g., subgroup analyses by socioeconomic status or other indicators) are not well reported in the abstract. Including the equity findings in the abstract may facilitate finding equity-focused reviews. As mentioned above, some readers only read or have access to the abstract. We felt that including equity-findings in the abstract will be helpful for users to determine whether the review is of interest. Therefore, the abstract should describe all relevant effects on health equity, both beneficial and harmful, as well as the methods used to assess health equity [35].

As recommended by PRISMA for abstracts [36], authors should report the main results in both numbers and words to meet the needs of different users.

*Item 2B: in addition, for equity-focused systematic reviews: Describe extent and limits of applicability to disadvantaged populations of interest.*

Example

- **○** “Conditional cash transfer programmes have been the subject of some well-designed evaluations, which strongly suggest that they could be an effective approach to improving access to preventive services. Their replicability under different conditions - particularly in more deprived settings - is still unclear because they depend on effective primary health care, and mechanisms to disburse payments. Further rigorous evaluative research is needed, particularly where [conditional cash transfers] CCTs are being introduced in low income countries, for example in Sub-Saharan Africa or South Asia.” [37]

Explanation - Since the abstract may be all that a reader accesses, it is important that the abstract reports the extent and limits of applicability of the findings of the review in relation to equity concepts. We felt this information is important to all consumers and users of the review, including patients, practitioners, policy makers, press, and the public.

The reporting of applicability is not intended to be a recommendation for practice or policy. It is instead intended to provide the reader with information regarding the primary studies and the results of the review and how the results of equity considerations apply. While there is insufficient space to report applicability considerations for all populations, we felt that applicability to the target population of the review should be reported.

### Introduction section

#### Item 3: rationale

*Standard PRISMA item: Describe the rationale for the review in the context of what is already known.*

*In addition, for equity-focused systematic reviews: Describe assumptions about mechanism(s) by which the intervention is assumed to have an impact on health equity.*

Examples

- **○** “CCT programs are justified on the grounds that demand-side subsidies are needed to address constraints and bottlenecks of service delivery. CCT programs usually aim to increase demand for preventive health services and education because these services have positive spillover effects that justify the expense. CCT help overcome barriers to access of services. These programs address social equity concerns because CCT can help to “level the playing field” thus creating equal opportunities” [37].
- **○** “Many lay health worker programs aim to address inequity by providing services to underserved communities” [38].

Explanation - If available, systematic reviews with a focus on health equity should explicitly describe the assumptions about the effects of the intervention on health equity, or drivers of health inequity. Assumptions about outcomes along the causal chain and these hypotheses about health equity may be articulated using different methods such as a program theory and can then be tested empirically with pre-planned analyses in the review [39, 40]. The review should describe *a priori* how and why interventions are expected to work and the influence of factors such as setting and participant and program characteristics. This explicit reporting of assumptions and underlying hypotheses will help the reader understand the choice of methods to assess effects on health equity and the interpretation of results within the framework of these hypotheses.

*Item 3A: in addition, for equity-focused systematic reviews: Provide the logic model/analytical framework, if done, to show the pathways through which the intervention is assumed to affect health equity and how it was developed.*

Examples

Figure 1 [41, 42]

**Fig. 1:**
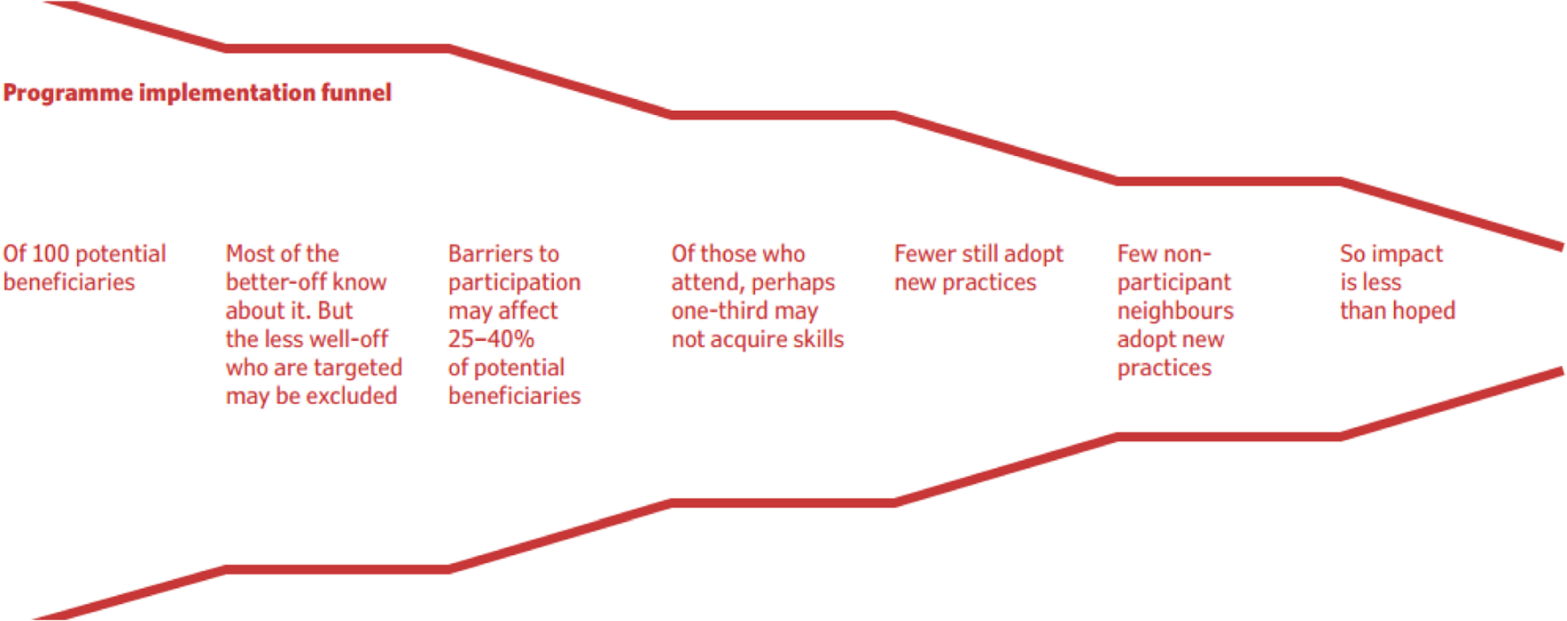
Item 3A, Example 1 – Analytic Framework. This is an example of a “funnel of attrition” [41, 42]

Figure 2 [43]

**Fig. 2:**
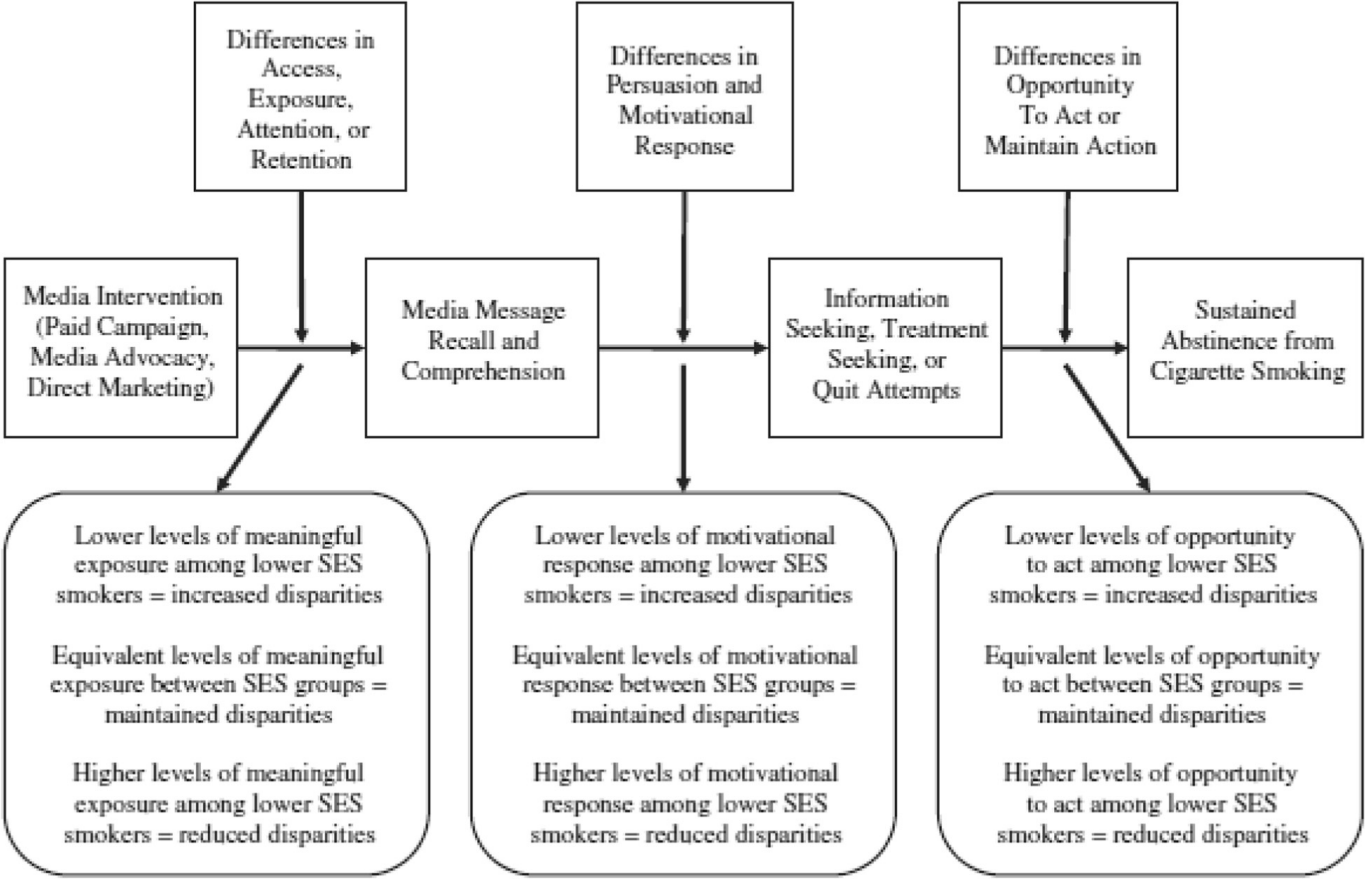
Item 3A, Example 2 – Analytic Framework. This is an example of a logic model [43]

Explanation - Health equity is influenced by multiple interacting factors such as context, setting, population characteristics, environment, public policy setting, health facility factors, health provider factors [4, 44, 45]. A visual framework, or logic model, can show the links between these factors, the program or intervention of interest, and the hypothesized effects on health equity. It can be used to show hypothesized pathways of influence on health equity, to focus the review, define the inclusion criteria, identify intermediate outcomes and harms, define the search strategy, and plan subgroup and effect modifier analyses [46]. For example, the logic model for a systematic review of preschool feeding shows that low socioeconomic status and household size may increase the risk of substitution (less food at home), thus decreasing the observed effects of preschool feeding) [47]. A visual framework can be useful for policy-makers and decision-makers who seek to answer questions about the effects of both targeted and universal interventions and how these programs will work in their policy/decision-making settings and system. For complex interventions, a visual framework can help the reader unpack the ‘black box’, thus showing how the intervention might affect different subgroups of the population and can be used as a tool for articulating subgroup analyses a priori.

Many different methods exist for developing a logic model, and different logic models have been developed for the same question. Guidance for how to construct a logic model is available from the Cochrane Public Health Group and the Kellogg Foundation [48–51].

#### Item 4: objectives

*Standard PRISMA item: Provide an explicit statement of questions being addressed with reference to participants, interventions, comparisons, outcomes, and study design (PICOS).*

*In addition, for equity-focused systematic reviews: Describe how disadvantage was defined if used as criterion in the review (e.g., for selecting studies, conducting analyses, or judging applicability).*

Example

- **○** “For the purposes of this review, the term’disadvantaged’ is taken to denote women whom the primary investigators considered to be of low socio-economic status or educationally disadvantaged, or who are under the age of 20[children born to teenage mothers in the UK have been estimated to have a 63 % increased likelihood of being born into poverty], or who are caring for children in single-parent households” [55].
- **○** “Parents with children up to the age of school entry and who were socially disadvantaged in respect of poverty, lone parenthood or ethnic minority status.” [17]
- **○** “We will retrieve studies implemented in LMICs, as defined by The World Bank Group’s classification… which study access to or utilization of PNC services by birthing women living in resource strained settings.” [33]

Explanation - Description of the specific population in the PICOS (Population, Intervention, Comparator, Outcome, Study Design) framework does not encompass explicit definition of how disadvantage or risk of inequity will be assessed, for example in reference to which group, disadvantaged by what mechanisms, and for which outcomes. Populations are at risk of health disadvantage for many different reasons which may interact with each other. There are many examples of factors that may contribute to disadvantage and these may interact with each other, such as geographical isolation, lack of access to health facilities, biologic vulnerability, historical oppression, social exclusion, health or language literacy, low resource settings, inadequate health systems, inadequate health insurance, health provider attitudes, stigmatization, and discrimination [6]. Systematic review authors can group such factors using the PROGRESS-Plus acronym; Place of residence, Race/ethnicity/culture/religion, Occupation, Gender/sex, Religion, Social Capital, Socioeconomic status [6, 52]. However, grouping populations that share one characteristic across PROGRESS-Plus may lead to overgeneralization since people within one category are heterogeneous (e.g., women range from poor to wealthy, with very different personal histories and exposures to health risks).

An explicit definition of how disadvantage will be assessed in the systematic review, either for targeted interventions or for subgroup analyses of universal interventions, is necessary to increase the likelihood that similar groups of people are compared, to make explicit the proposed reasons those people are considered disadvantaged, to explain why and how the program is expected to work for people at different risk of health disadvantage, and to facilitate judgments about applicability in different settings and populations. If disadvantage is used as a criterion, it needs to be defined, as well as the proposed reasons for disadvantage (e.g., biologic, societal), and the comparator or reference group against which disadvantage is assessed. Disadvantage and vulnerability may be poorly reported in primary studies. However, systematic review authors should describe how they have operationalized their definition of disadvantage or inequity in their inclusion criteria, analyses, and judgements of applicability. For example, if economically disadvantaged populations are the focus of the review, then a description of this population should be provided.

*Item 4A: in addition, for equity-focused systematic reviews: State the research questions being addressed with reference to health equity.*

Examples

- **○** 2 objectives “To determine the effectiveness of school feeding programs in improving physical and psychosocial health outcomes for low income school children.” And “To compare the effectiveness of school feeding programmes for socio-economically disadvantaged children and advantaged children” [53].
- **○** “To assess the impact on maternal and infant health and on infant development of programmes offering home support in addition to the standard service for teenaged mothers (ages less than 20 years) who had recently given birth and who were socially or economically disadvantaged, for example because they were poor, lived inner city or were single parents” [54].

Explanation - If assessing the impact on inequities is an objective of the review, the research questions related to this objective should be stated. Potentially important subgroup effects are differences in the relative effect that are large enough that users might make different decisions based on the subgroup effect than they would be based on the overall effect. Review authors should give consideration to all potentially disadvantaged groups for which the intervention might have a different effect based on the intervention’s mechanism of action; including economic status, employment or occupation, education, place of residence, gender, and ethnicity.

Consideration of differences in relative effects for disadvantaged populations should be addressed similarly to any other subgroup analysis. As such, authors should distinguish between protocol and review items. The protocol should indicate plans for any subgroup analyses, including specifying which subgroups will be investigated, the predicted direction of the subgroup effect, and the indirect evidence supporting the prediction (e.g., biological or sociological rationale; studies of other relevant populations, interventions or outcomes) [55, 56]. Only a small number of subgroups (i.e., only those for which there is a plausible reason such as indirect evidence for anticipating a subgroup effect) should be investigated.

### Methods section

#### Item 6: eligibility criteria

*Standard PRISMA item: Specify study characteristics (e.g., PICOS, length of follow up) and report characteristics (e.g., years considered, language, publication, status) used as criteria for eligibility, giving rationale.*

*In addition, for equity-focused systematic reviews: Describe the rationale for including particular study designs related to equity research questions.*

Examples

- **○** “Cross-sectional quantitative study designs, qualitative study designs, or a combination of the two (mixed-methods studies). Specifically, we included, first, any type of cross-sectional study design reporting quantitative data. Second, qualitatively-based studies had to have used either individual interviews or focus group interviews to collect data about [female genital mutilation/cutting] FGM/C and used qualitative data analysis methods, such as thematic analysis, to be eligible for inclusion. Third, mixed-methods studies that incorporated both quantitative and qualitative components where the research design matched the nominated study designs were included. Both the quantitative and the qualitative components of the study were subjected to the same inclusion criteria as the mono-methods studies and the study was only included when the inclusion criteria were met” [57].
- **○** “We included qualitative studies and studies using descriptive statistics which met the following criteria:
  1. reported on interventions as identified as “farmer field schools”, although not necessarily the same interventions as those included in the review of effects (review question 1);
  2. assessed determinants of service delivery quality, knowledge acquisition, adoption of technological improvements, diffusion, or sustainability (either directly or indirectly – for example, studies that were relevant to addressing barriers to and enablers of [farmer field schools] FFS effectiveness)…” [58]

Explanation - Evidence on equity impacts may come from a range of study designs, depending on the question, and the study designs included in a systematic review should be chosen based on the question according to their ‘fitness for purpose’ [59]. The evidence of effects may have been assessed using RCTs or other intervention study designs such as interrupted time series or controlled before-after study. However, for many equity-focused reviews examining social or public health interventions, the context in which the intervention operates is important and may be reported in qualitative studies [59]. Evaluations of policy-level and other interventions that have implications for reducing inequity and may provide important insight into the effects on equity may have been evaluated using non-randomised designs (e.g., natural experiments).

Authors should be able to capture different types of evidence through the inclusion of different study designs, but should justify inclusion of these designs and provide the rationale. For example, barrier and facilitator data collection and analysis often requires quantitative and qualitative data. New authors may not be aware of the rationale for preferred study designs. While most systematic reviews just list study design without rationale, the need to explain the rationale is not just an issue for equity, it should always be reported.

*Item 6A: in addition, for equity-focused systematic reviews: Describe the rationale for including the outcomes (e.g., how these are relevant to reducing inequity).*

Examples

- **○** “Other smoking-related outcomes included compliance with age-of-sale legislation, density of advertising and vending machines, brand appeal, and awareness and receptivity to antismoking campaigns. This broad range of smoking-related outcomes was included in order to encompass the diverse ways in which tobacco control policies can influence youth smoking-related outcomes” [60].
- **○** “Changes in equity of access - increased access for disadvantaged groups or a reduction in gaps in coverage – could also be an important outcome measure. This required a preliminary analysis and categorisation of the population of interest along a socio-economic scale. We accepted any relevant methodology (e.g., wealth/asset index) provided it was rigorous and described in detail” [37].

Explanation - As with all systematic reviews, outcomes need to be selected based on their relevance to the relevant stakeholder and/or user group (e.g., subjects/patients, practitioner (and the patient-practitioner dyad), the public, policy-makers and politicians). Equity-focused systematic reviews must, in addition, consider the relevance and importance of outcomes across categories of disadvantage that are deemed relevant for the review, including both health and non-health outcomes. Non-health related outcomes can have direct impacts on health and equity. For example, the Whitehall study found that employment grade levels are related to health differences in which people with lower grade jobs have higher rates of mortality and diseases, such as ischaemic heart disease, and lower self-perceived health status [61].

In addition, measures may need to be developed and/or adapted to ensure that the methodology does not disadvantage participation of bias results across populations. Other situations are often found when a measure may be used across a population, and where the intervention effectiveness is analysed according to cultural diversity within the population. For example, a systematic review of culturally appropriate health education assessed the influence of culturally adapted measurement tools on knowledge outcomes using sensitivity analysis [62]. The importance of outcomes for different settings and populations needs to be rated when selecting major outcomes, for example, in Summary of Findings (SOF) Tables for Cochrane reviews. A SOF table presents the main findings of the review for up to seven patient-important outcomes and rates the quality of the evidence [63]. SOFs are intended for those using the review, such as decision-makers [64].

Context, inconvenience and burden (e.g., financial burden) for populations need to be considered as potentially important outcomes in equity-focused reviews even if they are not commonly reported in primary studies. Financial burden may be relatively greater for those who are poor and other burdens, such as stigma or travel time, may be different for different populations.

Equity of access to care and coverage of health services are important outcomes for some interventions which seek to improve access. Horizontal equity implies equal health care for equal need, whereas vertical equity implies greater health care for greater need. Authors should take a pragmatic approach to assessing equity of access.

#### Item 7: information sources

*Standard PRISMA item: Describe all information sources (e.g., databases with dates of coverage, contact with study authors to identify additional studies) in the search and date last searched.*

*In addition, for equity-focused systematic reviews: Describe information sources (e.g., health, non-health, and grey literature sources) that were searched that are of specific relevance to address the equity questions of the review.*

Examples

- **○** “We chose to restrict our search of electronic databases to the 20 databases that had produced the highest yield in the search for a previous systematic review on a related topic, the health effects of new roads. We developed our search syntax iteratively. We first conducted a scoping search with a provisional set of terms, retrieved the 100 most relevant abstracts, and then added additional indexing or text word terms used in those references to our search strategy. We then adapted the search syntax for each database or interface used. We did not limit the search using terms for study design. We decided not to attempt a “systematic” internet search. Instead, we used three quality assured gateway sites...and our own knowledge to generate lists of potentially relevant web sites, from which we selected a purposive sample of 16 sites that contained bibliographies or searchable databases of documents. These represented a range of types of organisation (academic, government, and voluntary), countries of origin (Canada, all the countries of the European Union, Norway, and the United States of America), and language of publication (Danish, English, French, Norwegian, and Swedish)” [65].
- **○** “We searched the following electronic databases for primary studies:
  - The Cochrane Central Register of Controlled Trials (CENTRAL) 2009, Issue 1, part of the The Cochrane Library (www.thecochranelibrary.com) including the Cochrane Effective Practice and Organisation of Care (EPOC) Group Specialised Register (searched 3 March 2009)
  - MEDLINE, Ovid In-Process & Other Non-Indexed Citations and MEDLINE, Ovid (1948 to present) (searched 24 June 2011)
  - EMBASE, Ovid (1980 to 2009 Week 09) (searched 2 March 2009)
  - PsycINFO, Ovid (1806 to February Week 4 2009) (searched 4 March 2009)
  - EconLit, Ovid (1969 to February 2009) (searched 5 March 2009)
  - Sociological Abstracts, CSA (1952 to current) (searched 8 March 2009)
  - Social Services Abstracts, CSA (1979 to current) (searched 8 March 2009)
  - LILACS (searched 6 May 2009)
  - WHOLIS (searched 7 May 2009)
  - World Bank
  - Science Citation Index Expanded (SCI-EXPANDED) (1975 to present) (searched 8 September 2010)
  - Social Sciences Citation Index (SSCI) (1975 to present) (searched 8 September 2010). In addition we selected relevant databases from the LMIC database list at: http://epocoslo.cochrane.org. We did not search CINAHL or International Pharmaceutical Abstracts, so it is possible that studies relating to nursing or pharmaceuticals were missed. However, the general searches, including in websites focused on this topic, did not suggest that we had missed any relevant studies. We will add these databases when the review is updated” [66].

Explanation - Equity-focused reviews often go beyond issues of health and bridge other disciplines and thus information sources. For equity-focused systematic reviews, sources of information beyond the well-known health databases may be required. The search strategy may require inclusion of sources of information from different disciplines and different databases (e.g., sociological abstracts, IDEAhealth, non-health transportation or environmental content databases, and discipline-specific grey literature). Authors should describe all sources of information used for the search and provide a brief description of each, and justify why these information sources were considered necesary and appropriate.

Some relevant information may be available only to members of a certain association or working group. It would therefore be helpful for authors to report the accessibility of the sources of information in addition to website links or other information that may help the reader identify where the information has originated.

#### Item 8: search

*Standard PRISMA item: Present full electronic search strategy for at least one database, including any limits used, such that it could be repeated.*

*In addition, for equity-focused systematic reviews: Describe the broad search strategy and terms used to address equity questions of the review.*

Example

- **○** See Additional file 1: Table S2

Explanation - Authors of equity-focused systematic reviews should report the search strategy and search terms used to identify sources relevant to the equity questions. Equity questions may require comprehensive textword searches to identify specific populations, multi-component interventions or settings of interest which may require combinations of text words. Additionally, equity relevant reviews may relate to stigmatized populations, where language has evolved in order to identify the communities in a non-stigmatized way. Any search terms used should be clearly reported to ensure that the reader can duplicate the search. We do not suggest limiting the search to equity-relevant terms unless these equity search strategies have been validated. For example, the Cochrane Child Health filter has been validated [67]. Other validated search filters are collected in a repository by the InterTASC Information Specialists' Sub-Group Search Filter Resource. There is also ongoing work to validate a filter for identifying sex-specific analyses [68].

**Box 2:** A note about searching

Caution should be used when developing the search strategy. Limiting the search using equity-related search terms is not recommended as many studies are not indexed using equity-related terms and potentially relevant studies could be missed. For equity-focused reviews, the search strategy may need to be broadened to reduce the risk of missing potentially included studies. Review authors should plan more time for screening.	

#### Item 11: data items

*Standard PRISMA item: List and define all variables for which data were sought (e.g., PICOS, funding sources) and any assumptions and simplifications made.*

*In addition, for equity-focused systematic reviews: List and define data items related to equity, where such data were sought (e.g., using PROGRESS-Plus or other criteria, context).*

Example

- **○** “…extracted data on study design, description of the intervention (including process), details on participants (including age, sex, number in each group), length of intervention, definition of poor/low income, other socio-demographic variables, including place of residence, race/ethnicity, age, and nutritional status, critical appraisal (see below), physical, cognitive, and behavioural outcomes. We had planned to extract data on cost-effectiveness, but found none. Where possible, we recorded effects by socio-economic position.” [53]

Explanation - It is important for equity-focused systematic reviews to report all items for which data were sought even if the information was not available from the primary studies. Authors should explain the reasons for seeking data on these characteristics. If possible, authors should consider making their data extraction forms available online (e.g., as web-only appendices) or by request so that others may use or amend the forms in their own reviews.

Other data items that relate to the context of the population or intervention should also be reported as well as any interactions between context and PROGRESS-Plus factors. Each characteristic requires careful consideration regarding their definition and classification as well as their interaction with other contextual elements and how they influence health inequities. For example, there is no agreed system for classifying race, ethnicity, and culture, particularly across different countries [6].

PROGRESS-Plus is one acronym that can be used to describe disadvantage [6, 52, 69]. However, other frameworks for describing disadvantage and inequity exist and may also be used to capture equity-relevant data items. We support PROGRESS-Plus because it is easy to remember and is inclusive of all factors that may indicate disadvantage. About 68 % of systematic reviews describe the included population using one or more of the PROGRESS-Plus criteria and 13 % assess the effects of interventions disaggregated across one or more of these characteristics [24].

#### Item 14: synthesis of results

*Standard PRISMA item: Describe the methods of handling data and combining results of studies, if done, including measures of consistency (e.g., I*^2^*) for each meta-analysis.*

*In addition, for equity-focused systematic reviews: Describe methods of synthesizing findings on health inequities (e.g., presenting both relative and absolute differences between groups).*

Example

- **○**” Studies demonstrating an overall effect on anthropometric outcomes were initially categorized according to whether they were effective or not effective among lower SEP groups. Within these categories, we then analysed studies to identify common characteristics between interventions, including the degree to which they addressed structural barriers to behavioural change; as noted earlier, particular structural barriers may be more or less prevalent among different SEP groups in a population” [70].

Explanation - There is a need for clear and explicit reporting of choices regarding analyses about health inequity and their rationale *a priori.* This includes reporting what will be compared and how these comparisons will be made. There are over 20 different approaches available to measure health inequalities between two groups (e.g., rate ratio, rate difference, low to high ratio), or between more than two groups (e.g., slope index of inequality, concentration index, index of dissimilarity) [71]. Despite vigorous debate about the attributes, measurement properties and implications of different measures and choice such as the referent group, there is no single accepted measure of health inequalities, and all are subject to limitations [72]. Furthermore, the selection of how to measure health inequalities may bias the interpretation of results [71]. For example, the interpretation of any measure of changes in health inequalities over time depends on whether the outcome is an adverse effect or beneficial outcome, and on the baseline prevalence [73]. Authors should report the methods used to synthesize findings to ensure sufficient information.

Figure 3 [18]

**Fig. 3:**
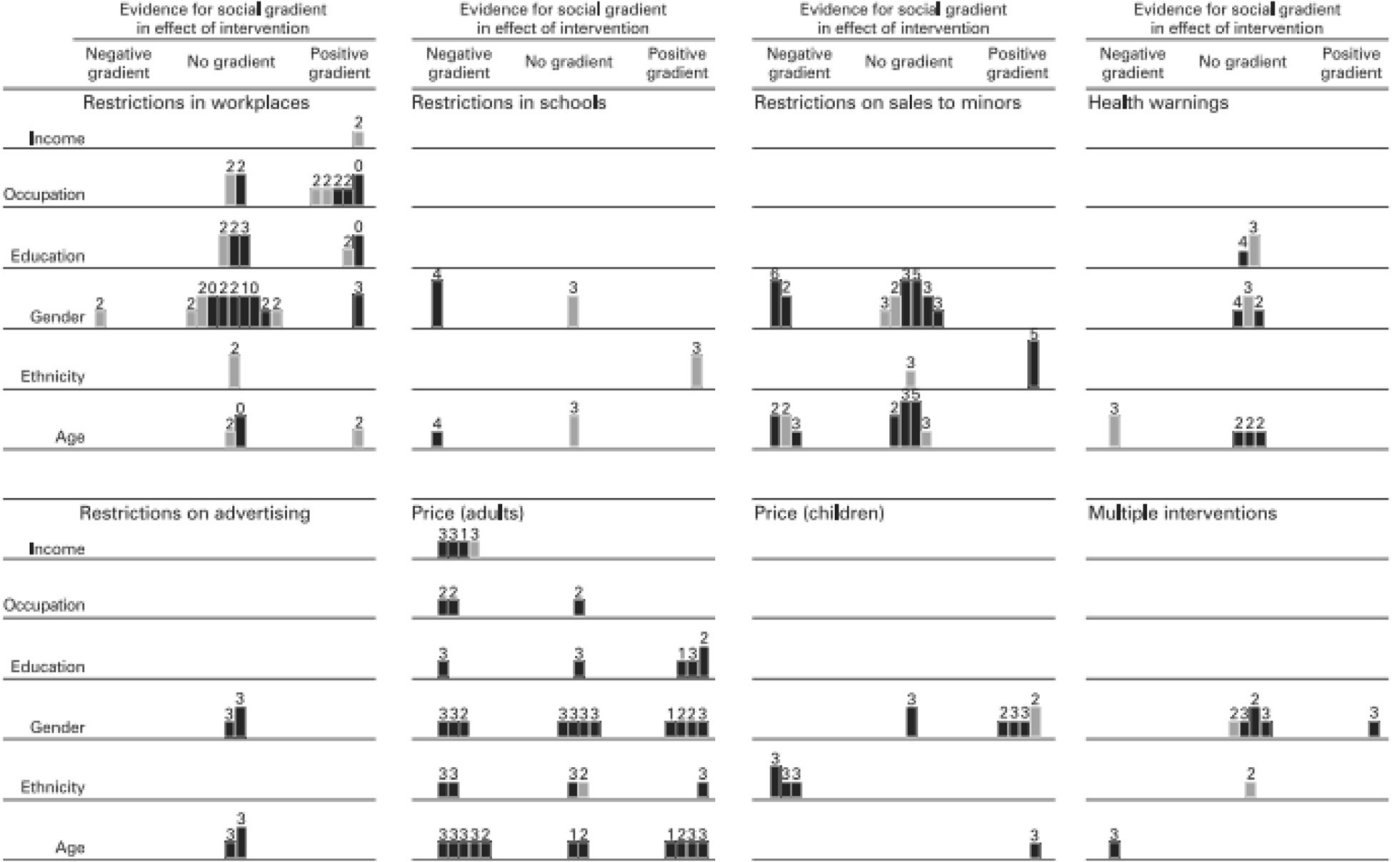
Harvest Plot. The ‘harvest plot’ synthesizes and displays the evidence to support possible social gradients in the effects of the intervention [18]

Measures of health inequalities may be useful as an input for population or economic models for projecting population impact on health inequalities. Considerations for choosing a measure of health inequalities are: 1) interpretability; 2) ease of calculation; and 3) information available from primary studies. While there is no agreement on the best approach, selection of measures of health inequalities needs to consider the advantages, data requirements and limitations of these approaches. The choice of reference point (comparator), method of assessing differences (relative or absolute), measurement of differences or end of study outcomes affects the difference observed between two or more groups [74]. Some measures (e.g., Gini, concentration index) may be less well understood by the users.

The measurement of health inequity depends on characteristics of the outcome measure and choices about comparisons, such as, whether the outcome is desirable or undesirable, baseline prevalence, and absolute or relative differences [74, 75]. This was demonstrated with a before-after study of a coronary artery bypass graft (CABG) report card program that compared the rates of CABG surgery between white, black, and Hispanic patients. The relative difference decreased between white and black patients for receipt of a CABG but the absolute difference increased therefore increasing the disparities between ethnicities [75, 76].

The absolute effect provides the difference in effectiveness between while the relative effect describes the difference in effectiveness relative to a reference group, such as the whole population [77]. Absolute differences can describe the proportion of the disadvantaged population affected, or not affected, by the intervention since disadvantaged populations may have worse health status and higher risk of adverse outcomes [75]. Another example (Fig. 2) demonstrates that while the rate of stomach cancer mortality for men and women declined between the years 1930 and 2000, the absolute difference between these rates decreased over that last 50 years while the relative difference has increased steadily [78]. This demonstrates that although mortality rates have declined in both groups, the ratio of male-to-female stomach cancer mortality has increased (more men than women are dying from stomach cancer). It would be misleading to present one of these indicators without the others therefore we suggest that systematic review authors present the absolute and relative differences.

If the aim of the intervention being studied in the systematic review is to reduce inequities, authors should report how they plan to measure the effect on health inequities. If the review will compare effects in two groups, how will the difference be measured, synthesised, and interpreted at the systematic review level?

Figure 4 [78]

**Fig. 4:**
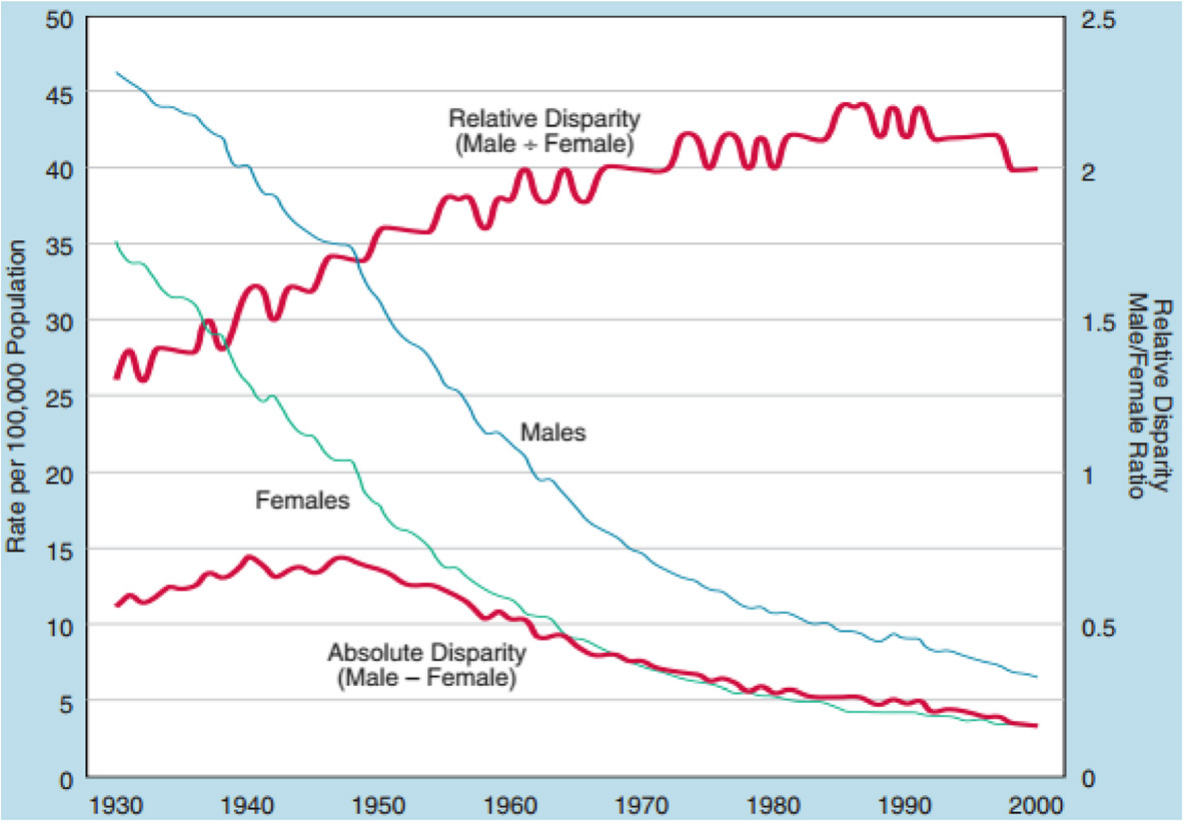
Absolute and Relative Gender Disparity in Stomach Cancer Mortality, 1930–2000 [78]

#### Item 16: additional analyses

*Standard PRISMA item: Describe methods of additional analyses (e.g., sensitivity or subgroup analyses, meta-regression), if done, indicating which were pre-specified.*

*In addition, for equity-focused systematic reviews: Describe methods of additional synthesis approaches related to equity questions, if done, indicating which were pre-specified.*

Examples

- **○** “Effect modifiers, such as high/low energy, compliance, substitution, and duration of the intervention were examined. In addition, study quality was considered since studies of lower quality often show higher effect sizes than those of higher quality. For example, biased outcome assessment is possible if the outcome assessors are not blinded to study group. This review tabulated the effects for each study by sorting them according to these effect modifiers (type of study, blinding versus unclear blinding, date of study, and high versus low energy) (Kristjansson et al. 2007). The effect of school feeding on learning outcomes may also be affected by contextual factors as teacher absenteeism and availability of learning materials, both of which may be worse in more disadvantaged communities.” [53]
- **○** “This study examined the influence of program implementation, program activities, program environment, and individual characteristics on welfare-to-work programs. The authors also considered the unemployment rate for each to determine whether the programs were affected by the availability of jobs in the area in which the program was implemented” [79].

Explanation - Understanding how to reduce health inequities may require additional quantitative or qualitative analyses, such as causal pathway analyses or process evaluations and a greater use of subgroup analyses to explore the differential effects of public health or population level interventions. Implementation of an intervention and its effectiveness may depend on participant characteristics such as age, education, gender, social status, context including the presence of complementary services, setting characteristics, and intervention characteristics (e.g., fidelity of intervention, delivery). Differences in participant characteristics, context and intervention design or delivery may limit the ability to conduct a meta-analysis. These characteristics of interventions, setting and participants may not be well-reported in primary studies, or have insufficient statistical power to find significant effects at the sub-group level [80, 81]. Systematic review authors may need to contact the primary study authors for information regarding subgroup analyses across different characteristics such as ethnicity and socioeconomic status. This information may have been analysed but not reported in the published papers [82, 83]. The move towards open access of trial data may make this more feasible in the future [84].

Subgroup analyses need to be conducted with caution and follow guidelines for reducing the likelihood of false results. Sun and colleagues have developed criteria for judging the credibility of subgroup analyses such as prespecifying the hypothesized direction of effects, using a test for interaction, and keeping the number of subgroup analyses few, and justified based on prior empiric evidence [55].

Systematic reviews with an equity focus should document and describe relevant and important characteristics of the participants and settings, as well as implications for the ability to conduct pre-planned analyses.

Numerous additional methods may be employed to assess the influence of contextual factors, participant characteristics and implementation such as qualitative review methods to understand the process of implementation and its relationship to effectiveness (e.g., using meta-ethnography, realist review or thematic analysis) [85]. When reporting the use of these methods, authors should use the relevant, method-specific reporting guidance to transparently report their methods (e.g., RAMESES reporting guidelines for realist review or the Cochrane Handbook extension on qualitative reviews) [86, 87].

### Results section

#### Item 18: study characteristics

*Standard PRISMA item: For each study, present characteristics for which data were extracted (e.g., study size, PICOS, follow-up period) and provide the citations.*

*In addition, for equity-focused systematic reviews: Present the population characteristics that relate to the equity questions across the relevant PROGRESS-Plus or other factors of interest.*

Examples - Present the population characteristics that relate to the equity questions across the relevant PROGRESS-Plus or other factors of interest.

- **○** “Of the 82 studies included in this review, 55 studies (67 %) were conducted in six high income countries: Australia, Canada, Ireland, New Zealand, the UK, and the USA. Forty-one of the 82 studies were conducted in the USA. Twelve studies (14.6 %) were conducted in eight middle income countries (Brazil, China, India, Mexico, Philippines, Thailand, Turkey, and South Africa). Fifteen trials (18.3 %) were from 10 low income countries Bangladesh, Burkina Faso, Ethiopia, Ghana, Iraq, Jamaica, Nepal, Pakistan, Tanzania, and Vietnam). In 59 studies the intervention was delivered to patients based in their homes. Five interventions were based solely in a primary care facility…A further eight studies involved a combination of home, primary care, and community based interventions. Four studies delivered the intervention mainly by telephone…while one implemented the intervention through community meetings. For five studies, other sites were used such as the workplace, churches, or homeless shelters” [38].
- **○** “Study participants had a mean age of 12.6 years and were described as of American Indian descent and representing the Pueblo, Navajo, Hopi, and Jicarilla Apache Indian Nations. The study setting was described as a boarding school exclusively for American Indian youth and promoting academic excellence” [88].

Explanation - Approximately 50 % of systematic reviews report the effect of sex/gender on outcomes, and less than 15 % report other PROGRESS-Plus characteristics which may be important [21, 24, 89]. PROGRESS-Plus information is reported in primary studies more often than in systematic reviews [21, 89]. Reporting the characteristics of populations associated with disadvantage, if relevant to the SR question, helps the user/reader compare their own setting and population to those included in the studies and can influence decision-making.

It may be useful to include criteria relating to judgements about which PROGRESS-Plus factors are relevant for the review question. A number of frameworks are available to help identify characteristics that are relevant in describing the socioeconomic and demographic characteristics of populations in addition to PROGRESS-Plus, such as SCRAP (Sex, Comorbidities, Race, Age and Physiopathology) [90]; and SUPPORT Collaboration framework [91]. There is little empirical evidence about the most efficient use of these frameworks. It is unlikely to be feasible to report all characteristics of interest, and not all characteristics may be relevant for each review. It is not necessary to report on all PROGRESS-Plus characteristics, as this might encourage data dredging. However, authors should consider which factors are relevant to their question *a priori*.

Authors should report characteristics of the setting, and whether these characteristics are entangled with the classification of disadvantage. For example, in the systematic review of school feeding for disadvantaged children, disadvantage was identified by attributes of the setting such as the location (poor, rural villages), the main occupation (e.g., subsistence farmers”) and the presence of school breakfast programs, which were only funded and provided in very poor areas with high malnutrition [53].

#### Item 21: synthesis of results

*Standard PRISMA item: Present results of each meta-analysis done, including confidence intervals and measures of consistency.*

*In addition, for equity-focused systematic reviews: Present the results of synthesizing findings on inequities (see 14).*

Example

- **○** “This review sought to identify studies which had reported on sociodemographic characteristics known to be important from an equity perspective. For this process, the PROGRESS (Place, Race, Occupation, Gender, Religion, Education, Socio-economic status (SES), Social status) framework was utilised. All studies reported the gender of participants at baseline. Four studies reported the race of participants and the level of education of parents…and two studies included information about the employment status of parents at baseline… included information on SES of participants at baseline based on parental income… reported some indicators related to place (the proportion of participating schools in a rural or urban region) and SES (the proportion of participating schools in an urban region which were also in an area considered to be underprivileged). When analysing data on outcomes, only three studies analysed results by any of the PROGRESS items. …analysed outcomes by gender…analysed outcomes by the same indicators of place and SES that were collected at baseline (these data are discussed above)” [20].

Explanation - Authors should report the results of all analyses related to health inequities and specify which analyses were determined *a priori* and which were conducted post hoc. Raw values, as well as absolute and relative effects on health inequities should be presented for the reasons discussed above. All analyses conducted at the review level should be reported, even if they were lacking in data or were not statistically significant.

When examining the data across a population to identify population subgroups experiencing disadvantage, interventions may have a greater absolute effect even if the relative effect is the same. For example, a cohort of women smokers found that the relative risk of coronary heart disease for cigarette smokers was slightly lower among women with hypertension, hypercholesterolemia, or diabetes than among those without them [92]. However, the absolute (or attributable) risk was two or more times higher for women with those conditions. Although the relative risk was lower, the absolute risk was much higher because the baseline risk of coronary heart disease was so much higher for non-smoking women with those conditions [92].

A summary of findings table is a recent requirement of Cochrane reviews which presents the main findings of the review and the quality of the evidence [63] and are intended for those using the review, such as decision-makers, and also facilitate the use of the review for developing guidelines and recommendations [64]. The Summary of Findings (SOF) table is recommended to include 7 patient-important outcomes. To appropriately consider equity using summary of findings tables, authors should consider three strategies: 1) include an outcome related to health inequity to show whether the intervention enhanced health equity (Table 2); [93]. 2) consider whether disadvantaged populations have different baseline risk of the important outcomes, and include separate row in the summary of finding table to show the absolute events for disadvantaged groups (Table 3); [94] and 3) Consider whether a separate Summary of Findings (SOF) table is needed because of expected differences in relative effects (Table 4) [95].

**Table 2:** Example of a summary of findings table that includes an outcome related to health inequity

The impact of user fees on access to health services in low- and middle-income countries				
Population: Anyone using any type of health service in low- and middle-income countries.				
Settings: Burkina Faso, Kenya, Lesotho, Papua New Guinea.				
Intervention: Introducing or increasing user fees				
Comparison: No fees				
Outcomes	Relative change in utilisation^1^	Number of studies	Quality of the evidence (GRADE)^a^	Comments
Equity outcome - health utilisation by quartile	N/A	1	⊕ ⊖ ⊖ ⊖ Very low^3^	This study where quality improvements were introduced at the same time as user fees found an increase in utilisation for poor groups. The authors did not report the results in a way that the relative change in utilisation could be calculated.

[93]

**Table 3:** Example of a summary of findings table that includes a separate row to show the absolute events for disadvantaged groups

Vitamin A supplementation for preventing morbidity and mortality in children from six months to five years of age						
Patient or population: Children aged between 6 months and five years						
Intervention: Vitamin A supplementation						
Comparison: Placebo or usual care						
Outcomes	Illustrative comparative risks^a^ (95 % CI)		Relative effect (95 % CI)	No. of Participants (studies)	Quality of the evidence (GRADE)	Comments
Diarrhoea-related mortality	Low risk population		RR 0.72; 95 % CI 0.57 to 0.91	90,951 (7 studies)	+++O moderate^2^	Total number of participants reflects number randomised to studies. The analysis combined cumulative risk and risk per/1000 years follow-up.
	3 per 1000^1^	2 per 1000 (2 to 3)				
Follow-up: 48–104 weeks	Medium risk population					
	4 per 1000^1^	3 per 1000 (2 to 4)				
	High risk population					
	9 per 1000^1^	6 per 1000 (5 to 8)				

[94]

**Table 4:** Example of a separate summary of findings table because of expected differences for disadvantaged population

	Positive	Neutral	Negative	Mixed	Unclear	Total
Increases in price/tax of tobacco products	14	6	4	1	2	27
Smokefree-voluntary, regional, partial	1	1	19	0	4	25
Smokefree-compulsory, national, comprehensive	2	9	6	1	4	19
Mass media campaigns	3	2	5	2	6	18
Mass media campaigns-quitlines and NRT	5	3	3	0	1	12
Controls on advertising, promotion and marketing of tobacco	2	7	0	0		9
Population-level cessation support interventions	4	2	0	1	2	9
Setting based interventions(community, workplace, hospital)	2	4	1	0	0	7
Multiple policies	0	2	0	1	1	4
Total policies	33	36	38	6	17	130^a^
Total studies	31^b^	30	37	6	14^b^	117

Summary equity impact of included studies and policies

^a^Eight studies assessed more than one type of pilocy Dinno 2009^35^ = Smokefree, Price/Tax; Frieden 2005^49^ = Smoke, Price/Tax, Multiple policies; Hawk ^99^ = Mass Media, Mass Media-quitlines and NRT; Hawkins 2012 ^53^ = Smokefree, Price/Tax

^b^Levy 2006^41^ = Smokefree, Price/Tax, Mass Media; Nagelhout 2013^54^ = Smokefree, Price/Tax Media; Scaap 2008^47^ = Smokefree, Price/Tax, Controls on advertising, promotion and marketing of tobacco, Multiple policies; Wilson 2010a^115^ = Controls on advertising, promotion and marketing of tobacco, Mass media-quitlines and NRT

[95]

#### Item 23: additional analyses

*Standard PRISMA item: Give results of additional analyses, if done (e.g., sensitivity or subgroup analyses, meta-regression [see Item 16]).*

*In addition, for equity-focused systematic reviews: Give the results of additional synthesis approaches related to equity objectives, if done, (see 16).*

Examples

- **○** “Effect modifiers were age and socioeconomic status. Younger students had larger effects than older students and students with lower socioeconomic status (SES) had larger effects than those with higher SES.” [96]
- **○** “This review used weighted regression analyses to investigate which elements of the programs were independently related to bullying and victimization effect sizes. These analyses showed that the most important elements of the program that were related to a decrease in bullying were parent training/meetings and disciplinary methods. Of all the intensity and duration factors, the most important program elements were intensity for children and parent training/meetings.” [97]

Explanation - The results of any additional syntheses related to the equity objectives should be reported as well as whether they were planned *a priori* and specified in the review protocol. This is consistent with published best practice in subgroup analysis [55]. Subgroup analyses can be inappropriate, poorly-specified, and prone to Type I and Type II error, therefore, aall subgroup analyses need to be interpreted cautiously. Subgroup analyses in systematic reviews are generally reported with insufficient detail to judge their credibility [56].

Subgroups that were not identified at the protocol stage may be identified post hoc, however, the rationale for these analyses should be reported. Authors should report all subgroup analyses and any analyses to assess effect modifiers such as meta-regression- both statistically significant and non-significant to avoid outcome reporting bias of reporting only statistically significant results [98]. This may be difficult, as effect modifiers may not be clearly reported in the primary studies. In some cases, there may be too few studies in particular settings of interest to draw conclusions. Intervention effects can be influenced by their design and implementation as well as the context within which it was implemented. For example, in the school feeding review, learning outcomes such as mathematics achievement were found to be higher with school meals programs but context was important for this outcome; if there were no teachers then there was no change in educational achievement with feeding.

Analyses related to contextual factors should be fully reported including a description of whether data was lacking from primary studies.

### Discussion section

#### Item 26: conclusions

*Standard PRISMA item: Provide a general interpretation of the results in the context of other evidence, and implications for future research.*

*In addition, for equity-focused systematic reviews: Present extent and limits of applicability to disadvantaged populations of interest and describe the evidence and logic underlying those judgements.*

Example

- **○** “This review included studies from high income countries as well as lower-middle- and upper-middle-income countries, with five studies conducted in countries within the latter two groupings (Thailand, Brazil, Chile and Mexico). This means that, while predominantly conducted within high-income settings, the findings from this review may be generalisable to a number of settings. A total of nineteen studies specifically reported incorporating strategies to target socio-economic and/or cultural diversity or disadvantage. One such study was conducted outside of the high-income country setting, in Chile, an upper-middle-income country. Of the remaining eighteen studies, seven studies conducted in the USA were of interventions targeting African American children and their communities and another two studies targeted Native American communities. Other studies targeted participants of low socio-economic status, or were implemented in areas of social disadvantage. By far the most common setting for interventions included in this review were schools (43 studies). Other interventions were (or included) home-based (14 studies), community based (six studies), or were set in a health service (two studies) or care setting (two studies). Eleven studies incorporated interventions across multiple settings.” [20]

Explanation - The conclusion should provide a transparent assessment of the applicability, the transferability, and the generalizability of the findings to the specific disadvantaged populations of interest (recognizing it is impossible to make these judgments for all possible populations). Authors should clearly report any disadvantaged population that was specified in the protocol and the reasons that consideration was given to the applicability of the results to the specified population. The applicability of the findings of a review to disadvantaged populations should be addressed similarly to considerations of applicability to any other population [8, 64, 90, 99, 100].

Authors should specify disadvantaged populations or settings for which the intervention is likely to be relevant. In the discussion review authors should consider the potential impact of: economic status, employment or occupation, education, place of residence, gender, and ethnicity as potential influences on the applicability of the results to disadvantaged populations, as well as resource or capacity constraints, health system arrangements or baseline conditions as potential reasons for there being a difference in the potential applicability of the results to low-income countries or disadvantaged populations. Authors should justify any judgments about applicability using transparent methods. There is no agreed checklist for judging applicability, though many are available [81, 101]. However, authors should provide rationale, and any data used to make judgements about applicability.

Applicability of results is often overlooked in systematic reviews. For example, an assessment of systematic reviews related to public health found that only 13 % discussed applicability [102]. The panel felt that the conclusion of an equity-focused systematic review should provide a transparent assessment of the applicability, the transferability, and the generalizability of the findings to at least one specific disadvantaged population of interest. This population should be pre-specified in the protocol with rationale. Authors should also specify additional disadvantaged populations or settings for which the intervention is likely to be relevant.

The applicability of the findings of a review to disadvantaged populations should be addressed similarly to considerations of applicability to any other population, using explicit methods [8, 64, 90, 99, 100]. There is no agreed checklist for judging applicability, though many checklists are available [81]. Authors should provide a rationale for the method they choose, and any data used to make judgements about applicability, such as other evidence about the possible impact of economic status, employment or occupation, education, place of residence, gender, and ethnicity, as well as resource or capacity constraints, health system arrangements or baseline conditions.

*Item 26A: in addition, for equity-focused systematic reviews: Provide implications for research, practice, or policy related to equity where relevant (e.g., types of research needed to address unanswered questions).*

Examples

- **○** “The body of evidence in this review provides some support for the hypothesis that obesity prevention interventions in children can be effective, and where examined, have not caused adverse outcomes or increased health inequalities. To this end, the direction of research and evaluation must move into how to implement effectively to scale, sustain the impacts over time and ensure equitable outcomes. In addition, interventions need to be developed that can be embedded into ongoing practice and operating systems, rather than implementing interventions that are resource intensive and cannot be maintained long-term.” [20]
- **○** “Future research should promote the development of effective interventions to enhance the online health literacy of consumers. Thus there is a need for well-designed and rigorously conducted randomised controlled trials (RCTs). These RCTs should involve diverse participants (regarding disease status, age, socio-economic group and gender) to analyse to what extent online health literacy reduces a barrier to using the internet for health information, or if socio-economic group, gender and age are more important in influencing internet use (Livingstone 2006). Trials should be conducted in different settings (including low, middle and high income countries) and should examine interventions to enhance consumers online health literacy (search, appraisal and use of online health information) like internet training courses.” [103]

Explanation - Implications for research, practice, and policy should highlight the effects on equity. This sections of the review should state the research that needs to be done to address existing knowledge gaps and should also suggest what the unanswered research questions are – that is, by specifying the questions that still need answering instead of stating that we “need more research”.

## Discussion

We developed the PRISMA-E 2012 checklist following guidance suggested by Moher and colleagues [23]. This reporting guideline is intended to improve transparency and completeness of reporting of equity-focused systematic reviews. Improved reporting can lead to better judgement of applicability by policy makers which may result in more appropriate policies and programs and may lead to reductions in health inequities.

This explanation and elaboration document is intended to accompany the PRISMA-E 2012 Statement to improve understanding of the reporting guideline for users [15]. The original PRISMA Statement has been endorsed by almost 200 journals; therefore, we recommend that authors of equity-focused systematic reviews use both the PRISMA checklist and the PRISMA-E 2012 checklist.

Potential limitations of the PRISMA-E 2012 checklist are that certain terminology used in the reporting guideline may not be well defined or widely used and may be defined differently by different users. To mitigate these concerns, we pilot tested the checklist with different groups of systematic reviews authors including those in high-income as well as low- and middle-income countries. The results of these pilot tests have been reported elsewhere [104]. While some of the PRISMA-E 2012 extension items may apply to non-equity-focused reviews, we felt that their importance for equity-focused reviews was great enough to warrant development of a specific reporting guideline for these reviews. In addition, there is no planned update of the PRISMA Statement so we have included them in this reporting guideline.

We are committed to a broad based dissemination strategy of PRISMA-E 2012 and hope to have endorsement by all journals endorsing the PRISMA Statement. Our dissemination strategy includes contact with journal editors, systematic review authors and trainers, and dissemination at meetings and conferences. We will continue to monitor endorsement of the checklist by journal editors. We plan to evaluate this reporting guideline at a future date to determine its impact on reporting of equity-focused systematic reviews. We will measure the ‘footprint’ of PRISMA-E 2012 by tracking the number of requests for support (e.g., emails, phone calls), and indicators of sharing of PRISMA-E 2012 through various networks, such as LinkedIn, Twitter, and Facebook. We will also measure web metrics, such as downloads of the Word file of the reporting guideline checklist from our website.

We hope that journal endorsement and implementation, and use by systematic reviewers will improve the reporting of equity-focused systematic reviews. Widespread use of the PRISMA-E 2012 checklist may increase the requests for more data from primary researchers which may in turn improve the reporting of equity considerations in primary research.

## Additional file

Additional file 1: Table S1. Systematic reviews with equity in the title (2013/11/28 to 2014/11/27). **Table S2.** Review of the research on the effectiveness of health service interventions to reduce variations in health. (DOC 35 kb)
