# Supplementary material for: Extending the PRISMA statement to equity-focused systematic reviews (PRISMA-E 2012): explanation and elaboration
Source: Int J Equity Health. 2015 Oct 8;14:92. doi: 10.1186/s12939-015-0219-2 (PMC4599721; doi:10.1186/s12939-015-0219-2)
Supplement: Additional file 1: Table S1. — Systematic reviews with equity in the title (2013/11/28 to 2014/11/27). Table S2. Review of the research on the effectiveness of health service interventions to reduce variations in health. (DOC 35 kb) [file 12939_2015_219_MOESM1_ESM.doc]

**Table S1: Systematic reviews with equity in the title (2013/11/28 to 2014/11/27)**

| **Query** | **Items Found** |
| --- | --- |
| **(equit*[ti] OR inequit*[ti]) AND (MEDLINE[Title/Abstract] OR (systematic[Title/Abstract] AND review[Title/Abstract]) OR meta-analysis[Publication Type])** | 11 |
| **(equit*[Title/Abstract] OR inequit*[Title/Abstract]) AND (MEDLINE[Title/Abstract] OR (systematic[Title/Abstract] AND review[Title/Abstract]) OR meta-analysis[Publication Type])** | 73 |
| **(MEDLINE[Title/Abstract] OR (systematic[Title/Abstract] AND review[Title/Abstract]) OR meta-analysis[Publication Type])** | 19885 |

### Table S2

Review of the research on the effectiveness of health service interventions to reduce variations in health

Medline [...]

Using CD Plus Ovid software, searching only from 1990 to December 1994 with no language

limitations and no publication type limitations.

***Group 2: Describing possible strategies to reduce variation in health***

011 consumer advocacy/

012 health promotion/

013 exp health planning/

014 insurance, health/

015 exp preventive health services/

016 exp health policy/

017 marketing of health services/

018 mass media/ 133

019 parenting/

020 primary health care/

021 exp public health/

022 exp self-help groups/

023 smoking cessation/

024 social support/

025 urban renewal/

026 exp public assistance/

027 health services accessibility/

028 delivery of health care/

029 environmental health/

030 exp patient acceptance of health care/

031 quality assurance, healthcare/

032 social change/

033 social justice/

034 public health/

035 accident prevention/

036 accidental falls/pc

037 accidents, home/pc

038 accidents, occupational/pc

039 accidents, traffic/pc

040 drowning/pc

041 consumer product safety/

042 disease outbreaks/pc

043 disease reservoirs/pc

044 disease transmission, patient-to-Professional/pc

045 disease transmission, Professional-to-patient/pc

046 environmental pollution/pc

047 health education/

048 hygiene/

049 sanitation/

050 insurance, medigap/

051 exp medicare/

052 medicare assignment/

053 medicaid/

054 ((free adj care) or ((nursery or preschool) adj education))).tw.

055 (after adj school adj care).tw.

056 (social adj support).tw.

***Group 3: Describing possible associations of variations in health***

057 housing/

058 public housing/

059 nutritional status/

060 age factors/

061 uncompensated care/

062 socioeconomic factors/ 134

063 social class/

064 single parent/

065 exp ethnic groups/

066 poverty/

067 poverty areas/

068 unemployment/

069 rural health/

070 rural population/

071 urban health/

072 educational status/

073 urban population/

074 urbanization/

075 exp homeless persons/

076 medically uninsured/

077 medically underserved area/

078 (underinsured or uninsured or uncompensated or indigen$ or (ethnic adj difference#)).tw.

079 (unemployed or unemployment or (low adj income#) or blacks or deprived or

deprivation).tw.

080 (disadvantaged or endowed or unendowed or education$ or equity or equitable).tw.

081 (inequity or inequities or inequitable or unequal or homelessness or illiterate).tw.

082 (inequality or inequalities or variation# or poverty or underprivileged).tw.

083 (family adj income) or (inner adj cit$) or (minority adj group#) or (vulnerable adj

group#)).tw. nutritional status/ or nutrition.tw.

084 minority groups/

085 [(((low or lower or less or poor or poorer or level) adj2(socieconomic or

education$)).tw.]

086 (workplace or ((manual adj worker#) or occupation#) or (blue adj collar)).tw.

***Group 4: Describing the characteristics potentially responsive to the intervention***

087 exp health services/ec,og,st,sd,td,ut

088 exp vital statistics/

089 (health adj effects).tw.

090 health services accessibility/

091 ((campaign# or program# or intervention#) adj4 (access or accessibility or utili#ation

092 uptake or effect#).tw.

(Arblaster et al 1996)
